# Diagnostic accuracy of WHO verbal autopsy tool for ascertaining causes of neonatal deaths in the urban setting of Pakistan: a hospital-based prospective study

**DOI:** 10.1186/s12887-015-0450-4

**Published:** 2015-10-05

**Authors:** Sajid Bashir Soofi, Shabina Ariff, Ubaidullah Khan, Ali Turab, Gul Nawaz Khan, Atif Habib, Kamran Sadiq, Zamir Suhag, Zaid Bhatti, Imran Ahmed, Rajiv Bhal, Zulfiqar Ahmed Bhutta

**Affiliations:** Department of Pediatrics & Center of Excellence in Women and Child Health, Aga Khan University, Karachi, Pakistan; Department of Pediatrics, King Edward Medical University, Lahore, Pakistan; Department of Child and Adolescent Health and Development, World Health Organization, Geneva, Switzerland; Center for Global Child Health, Hospital for Sick Children, Toronto, Canada

**Keywords:** Verbal Autopsy, Neonatal Death, Causes, diagnostic accuracy, Sensitivity, Specificity

## Abstract

**Background:**

Globally, clinical certification of the cause of neonatal death is not commonly available in developing countries. Under such circumstances it is imperative to use available WHO verbal autopsy tool to ascertain causes of death for strategic health planning in countries where resources are limited and the burden of neonatal death is high. The study explores the diagnostic accuracy of WHO revised verbal autopsy tool for ascertaining the causes of neonatal deaths against reference standard diagnosis obtained from standardized clinical and supportive hospital data.

**Methods:**

All neonatal deaths were recruited between August 2006 –February 2008 from two tertiary teaching hospitals in Province Sindh, Pakistan. The reference standard cause of death was established by two senior pediatricians within 2 days of occurrence of death using the International Cause of Death coding system. For verbal autopsy, trained female community health worker interviewed mother or care taker of the deceased within 2–6 weeks of death using a modified WHO verbal autopsy tool. Cause of death was assigned by 2 trained pediatricians. The performance was assessed in terms of sensitivity and specificity.

**Results:**

Out of 626 neonatal deaths, cause-specific mortality fractions for neonatal deaths were almost similar in both verbal autopsy and reference standard diagnosis. Sensitivity of verbal autopsy was more than 93 % for diagnosing prematurity and 83.5 % for birth asphyxia. However the verbal autopsy didn’t have acceptable accuracy for diagnosing the congenital malformation 57 %. The specificity for all five major causes of neonatal deaths was greater than 90 %.

**Conclusion:**

The WHO revised verbal autopsy tool had reasonable validity in determining causes of neonatal deaths. The tool can be used in resource limited community-based settings where neonatal mortality rate is high and death certificates from hospitals are not available.

## Background

Worldwide, an estimated 3 million neonatal deaths occur each year. Over the last two decades the proportion of neonatal deaths in the under-five deaths has increased from 37 % in 1990 to 44 % in 2012 [1]. Majority of the under five deaths are concentrated in only five countries of developing world, with Pakistan contributing approximately 6 % of total deaths [2]. Pakistan has high neonatal mortality rate 55 per 1,000 live births) compared to its neighboring countries; India, Bangladesh, Nepal and SriLanka [3]. There is paucity of data on causes of these neonatal deaths through the routine sources of information. In addition, majority of deaths in developing countries occur at home, and hospital based death certifications are not available [4]. Collecting accurate information on the causes of neonatal deaths has significant implications for planning and prioritizing of resources for such countries.

Historically in the developing world, to ascertain causes of neonatal deaths Verbal Autopsy (VA) tool has been employed [5]. The standard, World Health Organization (WHO) VA tool has acceptable sensitivity and specificity to ascertain causes of child deaths [6–9]. Unfortunately the same tool had poor diagnostic accuracy for neonatal deaths [6, 10–12]. Therefore the WHO formulated a specific tool to help resolve the quality issues for ascertaining causes of neonatal deaths [13]. A study undertaken In India found that this tool can provide reasonably good estimates of major causes of neonatal deaths in countries with high neonatal death burden [14]. This manuscript reports finding from a similar study conducted in Pakistan and adds up to the evidence base on the accuracy of the WHO neonatal VA tool in ascertaining cause of death in the developing world.

The objective of the study was to estimate the sensitivity, specificity, level of agreement and diagnostic accuracy of revised WHO verbal autopsy tool in ascertaining the cause specific mortality fractions (CSMF) for major causes of neonatal deaths in comparison with a reference standard cause of death assigned by physician. The physician diagnosis was determined by clinical history & examination, supportive radiology and laboratory data collected prospectively from health facilities.

## Methods

### Study setting

The study was conducted in two large public sector teaching hospitals located in the province of Sindh; The National Institute of Child Health in Karachi and Government Civil Hospital located in Hyderabad. These hospitals are tertiary care facilities that serve as a referral center for a significant population of Sindh and adjoining areas. Data was prospectively collected from August 2006 up to February 2008.

### Sample population and inclusion criteria

All neonatal deaths that occurred in the hospitals during the study period were included only if the families of the deceased resided within 100 km of the facility. Additionally, only those neonatal death for which physician had assigned the cause from available clinical information within 48 h were included in the sample

### Enrolment

**Fig. 1:**
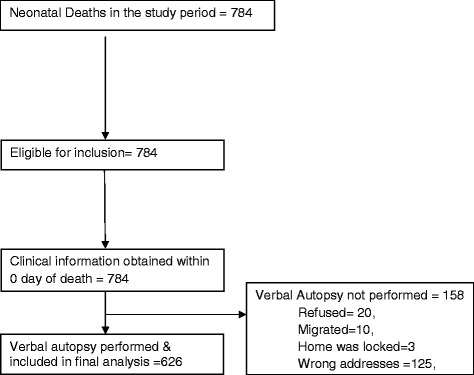
Flow Diagram for the Verbal Autopsy Enrolment

Figure 1 explains the enrolment process for this verbal autopsy study. During the study time period, 784 neonatal deaths were recorded in the participating hospitals and all were eligible to participate in the study. Verbal autopsy could not be performed in 158 cases; only 20 families refused an interview, 10 families had migrated, 3 homes were locked while 125 provided incorrect addresses. Therefore 626 cases were included in final analysis. The hospital records were considered as reference data and verbal autopsy data (verbatim) from community was used as the study data.

### Study tools

A newborn assessment form was developed to record details of maternal and newborn history. Information on antenatal, natal and post natal care, findings on newborn physical examinations and laboratory results was recorded upon admission in hospital. Daily clinical assessment of the newborn was documented in the follow up forms including events that evolved around death. This form was used to retrieve information to ascertain the reference cause of death through hospital records and subsequently to compile hospital based death certificate.

The World Health Organization (WHO)/ London school of tropical medicine and hygiene (LSTMH)/ Johns Hopkins University (JHU) modified verbal autopsy instrument (2000) was used for the evaluation of neonatal deaths. It was adapted to adjust cultural sensitivity and norms. The instrument had different sections for recording basic information about the deceased neonate and included narrative to record the respondent account of death. Additionally a section for disease related close ended questions on condition of newborn at birth, type of delivery, presence of any danger signs was also present. Instrument was translated into local language (Sindhi & Urdu) and back translated in English to ensure content validity. The instruments were pretested to identify problems or bottle necks that could arise during instrument administration. 

### Training of study staff

A six day’s training workshop was organized for community health workers (CHW’s) to train for administering the verbal autopsy interview and recording of information on the instrument. These training were undertaken by the study Investigators who were earlier trained as Master trainers by WHO experts on VA. There were 4 CHW’s in total, study supervisor and social scientist who received trainings. The training focused on interviewing techniques, and concepts used in the instrument. Objectives of the study and underlying meaning of the questions used in VA questionnaire were elaborated in a class room presentation and small group discussions. Audio visual aids were also used as per need. Simulated interview were conducted for practice in classroom followed by mock interviews at field site. These activities were closely observed by one of the study investigators. Feedback on the quality of simulated and mock activity was given to trainees on the same day on both of these activities. A 2 day refresher training session was arranged for the CHW’s every 6 months. This activity focused mainly on the revisions of the items indicated.

2 medical officers (already working as postgraduate students in same hospital) were trained for three days, in recording information of neonatal death from case record files (clinical, radiology and laboratory data) in the hospital on a standardized assessment form developed for the study.

**Fig. 2:**
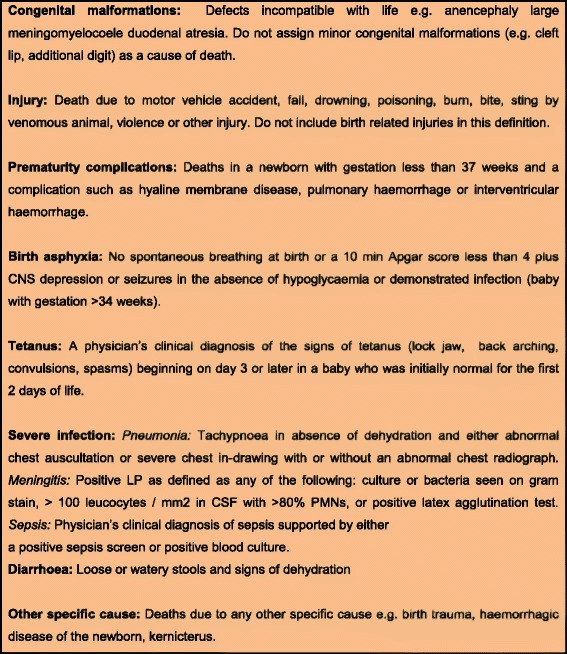
Cause of death; case definitions [12]

Similarly another three days training was arranged for the 4 study physicians. The physicians were Senior Pediatrician with significant experience in neonatal care working as faculty members in the department of Pediatrics and Child health at the Aga Khan University. They were trained on use of International classification of diseases, 10th version (ICD-10) [15] and assigning primary single cause (Fig. 2) as per hierarchy by NICE (Table 1) given in the study manual. The training was conducted by a WHO official. In order to standardize the assignment of primary cause of death, a standardized instruction manual for was developed and used across the study sites.

**Table 2:** Summary case review by physicians for hospital record and verbal autopsy

	Hospital record	Verbal autopsy
Reviewed cases by both reviewers	626	626
Consensus observed between both reviewers	494 (78.9)	461 (73.6)
Discrepant cases reviewed by third reviewers and finalized	132	165
Expert decision-Similar with any of the two reviewer	127	146
Expert decision-Different with both the reviewer	5	19
Causes of neonatal deaths similar in hospital and verbal autopsy	514 (82.1)	

### Ascertaining the reference cause of death from hospital records

Two trained medical officers (post graduates working in the two study hospitals) recorded the detailed course of events that led to neonatal deaths in the hospital. For all neonates admitted in the hospital clinical, radiology and laboratory data were recorded to help formulate a standard reference diagnosis. The maternal medical history, existing complications during pregnancy, details of antenatal care, labor, delivery along with newborn examination and detailed clinical information were recorded at the time of admission. The clinical case sheets were then checked for completeness and errors by study supervisor/principal investigator.

This information along with standard clinical definitions was used by two qualified postgraduate pediatricians with extensive experience to assign cause of death in hospital based death certificate. These two reviewers were kept independent and blinded to each other assessments and diagnosis. In case of disagreement between the two reviewers, the record was reviewed by a third senior pediatrician who served as an arbiter and the cause of death on which two of the three reviewers agreed was assigned. Incase all three had differed on single cause then it would have been labeled as “unclassifiable” [15].

### Assignment of cause of death from verbal autopsy

The verbal autopsy interviews were performed 2 to 6 weeks after the newborn death. In light of past experience with VA data collection this window to collected data was designed to allow for the family a grievance period to mourn the dead. We ensured that the period comply with the cultural norms. Trained female CHW’s, with an education level ranging from college graduates and above conducted the verbal autopsy interview at home. The mother was the primary respondent; however in some cases a female family member present at delivery and during newborn illness was also interviewed. However, the health care provider who attended the birth was not interviewed for the verbal autopsy. If the respondent was not available on the first visit, a repeat visit was made within the 2–6 weeks window. Written informed consent in the local language was obtained prior to interview. During the interview, pictorials of major congenital malformations and, low birth weight babies were shown to aid recall.

Two independent study physicians who were not involved in the care of the newborn and were trained in VA tool assigned the cause of death by using standardized case definition and list of causes of neonatal deaths (Fig. 2).

The two trained physicians independently reviewed the completed verbal autopsy tool. They were blinded to each other. In case of disagreement between the two, a third senior study physician who served as an arbiter reviewed the same case and the cause on which two of the three agreed was assigned. Incase all three had differed on single cause then it would have been labeled as “unclassifiable”. Primary and secondary associated causes of neonatal deaths were coded; primary cause of death was analyzed.

### Ethical clearance

The study was approved by Ethical Review Committee of Aga Khan University and Institutional Review Board (IRB) of WHO. Written informed consent was sought from each verbal autopsy respondent before inclusion into the study. Confidentiality of data was maintained throughout the study and was only accessible to the senior project staff. Participants in the study were allocated unique ID number for identification.

### Quality assurance

The quality of data was ensured through review meetings and supervisory field visits. A random 5 % of verbal autopsy interviews were also attended by the study supervisor. The purpose of these visits was to ensure if correct interview procedure and probing techniques were being applied by the interviewers. Additional 2 % work of each VA field interviewer was verified by directly by Social Scientists through blind re interviews to ensure that the data collected by the VA field interviewer is correct, real and contains minimum bias. Daily progress report was generated by the data management unit and the supervisor conducted daily debriefing meetings for problems pertaining to interviews and operations. Random field visits were undertaken by study investigators and WHO associates to ensure adequacy of verbal autopsy procedures both in hospital and in the community.

### Data management and analysis

Data was processed using the Visual FoxPro data management software (Fox Pro v 6.0 Microsoft Corp Seattle WA USA). Data entry was done using a standardized database structure. The database and range and consistency checks were prepared centrally with inputs from all sites. The verbal autopsy interview forms were double checked for completeness by supervisor before data entry. Range and internal consistency checks were performed regularly. All the data was double entered. To assess diagnostic accuracy of verbal autopsy we used sensitivity, specificity, positive predictive value (PPV) and negative predictive value (NPV) and their 95 % confidence intervals (CI) for leading causes of neonatal deaths. Verbal autopsy diagnoses were compared with the reference diagnoses using simple chi sq analyses. Sensitivity ±10 % precision and specificity ±5 % precision determined compared to the reference standard for all diseases

## Results

Figure 1 illustrates the status of enrolments in the verbal autopsy study. Overall, 784 neonatal deaths were recorded during the study period. Verbal autopsy could not be performed in 158 cases of which only 20 refused for interview, 10 shifted from their homes (migrated), 3 houses were found locked and 125 gave incorrect address. Verbal autopsies were successfully completed in 626 cases which were included in final analysis.

Table 2 provides a summary of death cases review by hospital record as well as verbal autopsy. Hospital records for all 626 cases were reviewed. Consensus observed between both reviewers for ascertainment of cause of death from hospital records was on 494 (78.9 %) cases while for 132 cases third reviewer was consulted. In 127 cases consensus was observed between the third and any of the first two reviewers and there were only 5 cases where all the three reviewers had assigned a different cause of death. Similarly for 461 (73.6 %) verbal autopsy cases consensus was observed amongst the two reviewers, however for 165 cases third reviewer was consulted. In 146 cases the third reviewer decision concurred with any of the first two reviewers however in 19 cases were labelled as unclassified as all the three reviewer had different opinions. In 82 % of cases there was consensus amongst clinical diagnosis and verbal autopsy for causes of death.

**Table 3:** Baseline characteristics

Maternal characteristics	[*N* = 626]
Age of the mother (years), mean [SD]	28.1 [5.2]
Education (years), mean [SD]	8.5 [3.0]
Gestation age (weeks), mean (SD]	33.6 [4.1]
Multiple births, *n* [%]	99 [15.8]
Neonatal characteristics	
Birth weight (grams), mean [SD]	2398.6 [1578.4]
Age of the neonates in days at admission, mean [SD]	3.1 [5.6]
Mean age at death (days), mean [SD]	5.9 [6.7]
Male, *n* [%]	373 [59.6]
END (0–7 days), *n* [%]	446 [71.2]
LND (8–28 days), *n* [%]	180 [28.8]
Low birth weight (<2500 grams), *n* [%]	328 [64.2]
*N*	511
Preterm births (<37 weeks), *n* [%]	380 [68.1]
*N*	558

### Basic characteristics of neonatal deaths

Table 3 shows characteristics of mothers and neonatal deaths. Mean age of mother was 28 years, while level of education was 8.5 years. Gestational age was only known for 558 mothers and 68 % births were found to be preterm (<37 weeks). The enrolments were balanced in terms of gender and 60 % of the newborns were male. Out of the 626 deaths included in the final analysis birth weight data was only available 511 newborns. 328 (64 %) newborns were low birth weight (<2500 g). The mean age on the day of hospital admission was 3 days and the mean age at the time of death was 5.9 days. Majority of the deaths (71 %) occurred within the first week of life. Unexplained neonatal death was 17 (2.7) and 16 (2.6) in clinical and verbal autopsy review respectively, others specific causes were 11 (1.8) & 6 [1].

**Table 4:** Cause specific mortality fraction for neonatal deaths as per clinical and verbal autopsy diagnosis

Cause of neonatal deaths	Clinical diagnosis, *n*(%) [*N* = 626]	Verbal autopsy, *n*(%) [*N* = 626]
Congenital malformations	14 (2.2)	13 (2.1)
Prematurity [<33 wks]	224 (35.8)	229 (36.6)
Birth asphyxia	176 (28.1)	188 (30)
Tetanus	9 (1.4)	9 (1.4)
Pneumonia	11 (1.8)	13 (2.1)
Meningitis	1 (0.2)	3 (0.5)
Diarrhea	1 (0.2)	0 (0)
Sepsis	162 (25.9)	149 (23.8)
Unexplained neonatal death	17 (2.7)	16 (2.6)
Others specific cause	11 (1.8)	6 (1)

### Cause specific mortality fractions

Table 4 presents the cause specific mortality fractions as per clinical and verbal autopsy diagnosis. Prematurity (<33 weeks) was found to be the leading cause of deaths according to both clinical (36 %) and verbal autopsy diagnosis (37 %). Second most frequent cause of death in this cohort as per clinical diagnosis (28 %) and verbal autopsy (30 %) was found to be birth asphyxia. Sepsis was the third common cause of death in light of clinical (26 %) and verbal autopsy diagnosis (24 %).

**Table 5:** Sensitivity and Specificity of verbal autopsy against clinical diagnosis

Cause of neonatal deaths	True positive	False positive	True negative	False negative	Sensitivity		Specificity		PPV		NPV	
	value	value	value	value	%	[95 % CI]	%	[95 % CI]	%	[95 % CI]	%	[95 % CI]
Congenital malformations	8	5	607	6	57.1	32.6–78.6	99.2	98.1–99.6	61.5	35.5, 82.3	99.0	97.9, 99.5
Immaturity [<33 wks]	209	20	382	15	93.3	89.2–95.9	95.0	92.4–96.7	91.3	86.9, 94.3	96.2	93.8, 97.7
Birth asphyxia	147	41	409	29	83.5	77.3–88.3	90.9	87.8–93.2	78.2	71.8, 83.5	93.4	90.6, 95.3
Tetanus	6	3	614	3	66.7	35.4–87.9	99.5	98.6–99.8	66.7	35.4, 87.9	99.5	98.6, 99.8
Severe infection^a^	138	27	424	37	78.9	72.2–84.3	94.0	91.4–95.9	83.6	77.2, 88.5	92.0	89.1, 94.1

^a^Severe infection included Sepsis, Meningitis, Pneumonia & Diarrhea

*PPV* Positive Predictive Value

*NPV* Negative Predictive Value

Other causes include congenital malformations, pneumonia, tetanus; meningitis diarrhea, unexplained deaths and other specific causes. Cause specific mortality fractions were comparable for hospital records and verbal autopsy. Similarly there was a general consensus between VA tool and standardized clinical and supportive data in ascertaining the causes of neonatal deaths.

### Sensitivity and specificity of verbal autopsy against clinical diagnosis

The results of sensitivity, specificity, positive predictive value (PPV), negative predictive value (NPV) = for five leading causes of neonatal deaths are shown in Table 5. The observed sensitivity and specificity values for clinical diagnosis and VA technique across the five leading causes of neonatal deaths varied from (57.1–93.3 %) and (90.9–99.5) respectively.

**Table 1:** Hierarchy for assigning primary cause of neonatal death [25]

Hierarchy of the cause of death (to be assigned in this order if criteria are met)	Age at death <3 days and gestation <32 weeks	Age at death ≥3 days and gestation <32 weeks	Age at death <3 days and gestation ≥32 weeks	Age at death ≥3 days and gestation ≥32 weeks
1	Congenital anomalies	Congenital anomalies	Congenital anomalies	Congenital anomalies
2	Injuries (not birth related)	Injuries (not birth related)	Injuries (not birth related)	Injuries (not birth related)
3	Asphyxia^a^	Asphyxia^a^ (if age < 7 days)	Asphyxia^a^	Asphyxia^a^ (if age <7 days)
4	Prematurity complications	Tetanus	Serious infection	Tetanus
5	-	Serious infection	Prematurity complications	Serious infection/diarrhoea
6	-	Prematurity complications	Other specific cause	Prematurity complications
7	-	-	-	Other specific cause

^a^It may be difficult to assign asphyxia as the primary cause of death in premature babies <34 weeks gestation (i.e. the baby did not breathe at birth due to prematurity) An alternative is to that asphyxia be collapsed into the prematurity complications if gestation is less than 34 weeks

Of the 626 neonatal deaths, 93 % prematurity and 83.5 % birth /perinatal asphyxia related deaths were correctly diagnosed by VA. The specificity for diagnosing deaths due to prematurity and birth /perinatal asphyxia was 95 % and 91 % respectively. Verbal autopsy technique has the least sensitivity for diagnosing deaths due to congenital malformation 57 %.

## Discussion

Our study showed prematurity, perinatal asphyxia and sepsis as the leading cause’s accounting for more than 90 % of all newborn deaths which is quite comparable to global estimates [1]. Verbal autopsy tool proved to have an acceptable level of accuracy in diagnosing leading causes of neonatal deaths. The high level of sensitivity of VA in diagnosing neonatal death due to prematurity signifies high accuracy standards of the new tool.

The specificity for all neonatal deaths in our study remained above 90 % while the sensitivity ranged from 57 % to 93.3 %. The lower sensitivity for congenital anomalies 57 % highlights the limitation of VA tool for this particular cause of neonatal mortality. However this may well be due to both lack of specific description of the anomalies in our settings and also the presence of concealed anatomical malformations such as cardiac and certain brain anomalies. Although the sensitivity level for congenital malformations was lowest (57 %) but it was above the acceptable level and slightly higher than the figure reported from India (33 %) [14].

Our results are consistent with other studies that used the WHO verbal autopsy tool to ascertain causes of neonatal deaths [1, 16] and the reported sensitivity are above the acceptable range for accurately diagnosing neonatal cause of death [17].

The proportion of neonatal deaths in our study was higher in male (59.6) and the possible reason for higher mortality rates in males may be the greater care seeking behaviors for male gender [3]. This social behavior underscores the need for robust behavioral change communication strategies to overcome the gender inequity that prevails in our society especially in periurban and rural areas.

The three leading cause specific mortality fraction (CSMF), prematurity birth asphyxia and sepsis reported in our study are comparable [18]. The consistency of our findings with global causes of neonatal deaths provides indirect evidence of the reliability of the WHO VA tool in estimating cause of death at a population level as well as the adequacy of the sample size. However prematurity came out as the leading cause of death. The numbers may have been overestimated in our study due to the use of last menstrual period (LMP) date method for gestational age calculations The LMP method was considered for our study due to lack of other cheap and reliable methods for confirmation of gestation. In the developing countries majority of women deliver without undergoing antenatal visits and ultrasound assessments. Therefore accurate assessment of gestational age is usually difficult and an overestimation is much more likely.

Our study had several strengths. It was one of the largest well designed prospective validation study for neonatal death in the region. We had sought the services of two well qualified post graduate (FCPS, FRCP) expert Pediatricians with more than 10 years of clinical experience to review the available information in hospital records including death certificate for all neonatal deaths and assigned a reference standard primary cause of neonatal death in the light of ICD-10. The two verbal autopsy reviewers had received extensive training by WHO expert trainer in assigning the cause of death and following case definitions. They worked independently and were blinded to each other in determining the cause of death. Furthermore, a standardized instruction manual for guiding physicians in the assignment of cause of death was developed and used across the study sites. The study physician coded for both the primary and underlying cause of death, but only primary cause was analyzed for this paper.

### Limitations

We enrolled neonatal deaths from two urban hospitals which may not be the representative of population at risk of the entire country. We used physician reviews for assigning the VA cause of death which is the most commonly used method although the results may vary considerably. [19] One of the disadvantages of this method is the lack of objectivity and inter-observer variability which we addressed in our study by providing standard objective case definitions and hierarchical causes of death and extensive training to the physicians reviewing verbal autopsy interviews. Additionally, the method is labor intensive and costly and therefore challenging to use in routine monitoring of causes of death, such as from Sample Registration Surveys in India and China.[17, 20]. The advantages include a contextual and holistic view of the historical data and which helps develop case history and causal pathway. An interesting alternative is the use of pre-decided computer algorithms. Recently computer algorithms have also come under some criticism and despite of all limitations, physician reviews are still considered the more reliable and accurate [21] (We found WHO verbal autopsy tool for neonatal deaths very effective, easy to use and the case definitions simple and applicable. Perhaps this was one of the reasons that the number of unexplained neonatal death was low in our study. Accuracy of verbal autopsy tool in determining major causes of deaths is dependent on obtaining a suitable reference diagnosis. Numerous studies in developing countries have used causes of death based on medical records as the “gold standard” [7, 22–24]). The limitations of medical records as “gold standard” needs to be recognized and acknowledged as there are instances in which the case notes in hospital record may be incomplete and availability of relevant investigation lacking. Physician diagnosis based on medical records may or may not be supported by relevant diagnostic tests, and can affect the accuracy of the “gold standard”. In settings where diagnostic modalities are limited and health information system solely depends on hospital reports; verbal autopsy would serve as a useful adjunct tool for determining the cause of death till the process of vital registration is comprehensively in place.

The data for the VA interview was collected between the 2–6 weeks after the neonatal death. This limit was defined in light of past experience with VA data collection. The window allows for the family a grievance period to mourn the dead. We ensured that the period comply with the cultural norms. We assume that this period could not be the cause for recall bias as events like deaths and the intermediate circumstances leading to death are known to be remembered.

## Conclusion

Our findings suggest that the WHO revised verbal autopsy tool has reasonable validity in determining causes of neonatal deaths in Pakistan. The WHO verbal autopsy tools can be used in resource limited community-based settings where neonatal mortality rate is high and death certificates specifying cause of death from hospital are not available.
